# Impact of Western Diet on Enterohemorrhagic *Escherichia coli* Colonization in the Human *In Vitro* Mucosal Artificial Colon as Mediated by Gut Microbiota

**DOI:** 10.3390/nu16132046

**Published:** 2024-06-27

**Authors:** Deborah O’Sullivan, Trisha Arora, Claude Durif, Ophélie Uriot, Morgane Brun, Marc Riu, Elisabet Foguet-Romero, Iris Samarra, Xavier Domingo-Almenara, Cormac G. M. Gahan, Lucie Etienne-Mesmin, Stéphanie Blanquet-Diot

**Affiliations:** 1 UMR 454 INRAe, Microbiology, Digestive Environment and Health (MEDIS), Université Clermont Auvergne, 28 Place Henri Dunant, F-63000 Clermont-Ferrand, France; deborah.o_sullivan@uca.fr (D.O.); claude.durif@uca.fr (C.D.); uriot.ophelie@hotmail.fr (O.U.); morgane.brun@outlook.fr (M.B.); lucie.etienne-mesmin@uca.fr (L.E.-M.); 2 Centre for Omics Sciences (COS), Unique Scientific and Technical Infrastructures (ICTS), Eurecat—Technology Centre of Catalonia & Rovira i Virgili University Joint Unit, 43204 Reus, Spain; trisha.arora@eurecat.org (T.A.); marc.riu@eurecat.org (M.R.); iris.samarra@eurecat.org (I.S.); xavier.domingoa@eurecat.org (X.D.-A.); 3 Department of Electrical, Electronic and Control Engineering (DEEEA), Universitat Rovira i Virgili, 43007 Tarragona, Spain; 4 Computational Metabolomics for Systems Biology Lab, Eurecat-Technology Centre of Catalonia, 08005 Barcelona, Spain; 5 APC Microbiome Ireland, University College Cork, T12 YT20 Cork, Ireland; c.gahan@ucc.ie; 6 School of Microbiology, University College Cork, T12 K8AF Cork, Ireland; 7 School of Pharmacy, University College Cork, T12 K8AF Cork, Ireland

**Keywords:** enterohemorrhagic *Escherichia coli*, colonization, Western diet, *in vitro* gut model, gut microbiota, mucus, short-chain fatty acids, bile acids

## Abstract

Enterohemorrhagic *Escherichia coli* (EHEC) is a major food-borne pathogen that causes human disease ranging from diarrhea to life-threatening complications. Accumulating evidence demonstrates that the Western diet enhances the susceptibility to enteric infection in mice, but the effect of diet on EHEC colonization and the role of human gut microbiota remains unknown. Our research aimed to investigate the effects of a Standard versus a Western diet on EHEC colonization in the human *in vitro* Mucosal ARtificial COLon (M-ARCOL) and the associated changes in the gut microbiota composition and activities. After donor selection using simplified fecal batch experiments, two M-ARCOL bioreactors were inoculated with a human fecal sample (*n* = 4) and were run in parallel, one receiving a Standard diet, the other a Western diet and infected with EHEC O157:H7 strain EDL933. EHEC colonization was dependent on the donor and diet in the luminal samples, but was maintained in the mucosal compartment without elimination, suggesting a favorable niche for the pathogen, and may act as a reservoir. The Western diet also impacted the bacterial short-chain fatty acid and bile acid profiles, with a possible link between high butyrate concentrations and prolonged EHEC colonization. The work demonstrates the application of a complex *in vitro* model to provide insights into diet, microbiota, and pathogen interactions in the human gut.

## 1. Introduction

Enterohemorrhagic *Escherichia coli* (EHEC) is a food-borne pathogen responsible for human diseases ranging in severity from diarrhea to hemolytic uremic syndrome [1,2,3]. EHEC serotype O157:H7 has emerged as the most virulent out of over 400 serotypes that have been identified, responsible for sporadic cases and outbreaks via the ingestion of contaminated food and water [4,5]. EHEC’s pathogenicity mainly relies on Shiga toxin production; however, other factors seem to play an important role in the pathogen’s survival, such as host age and intestinal microbiota composition [6]. Relatively little data are available on the interactions between EHEC and the human gut microbiota in a healthy state [7,8], but even less information is available for a diseased state or in the context of at-risk situations associated with a perturbated gut microbiota [7]. Diet has a considerable influence on human gut microbiota composition and metabolic activities [9]. The Western diet, characterized in particular by a high fat and carbohydrate content but low fiber intake, has been shown to induce alterations in the human gut microbiota that have been linked to the obesity epidemic and could promote various chronic inflammatory diseases [10,11,12,13]. A decrease in nutritional diversity, particularly a high-fat/low-fiber diet, has been associated with a drop in gut microbial diversity [14], which can further impact the abundance of short-chain fatty acid (SCFA)-producing bacteria [15,16].

Alterations in gut microbiota induced by the Western diet can in turn influence the susceptibility to enteric disease [17], as previously shown for the Adherent and Invasive *Escherichia coli* (AIEC) pathotype in mice [9]. A Western diet altered the gut microbiota composition and dynamics of *Citrobacter rodentium*, which is a murine model of EHEC infection; the most striking consequence of Western diet consumption was the frequent inability of mice to clear this pathogen [10]. A lack of dietary fiber resulted in increased digestion of the mucosal layer by gut microbiota, leading to a reduction in the protective effect of this physical barrier against *C. rodentium* in mice [18]. Lastly, dietary fiber content has been shown to affect the susceptibility to *E. coli* O157:H7 infection in mice, since some results suggested that individual diet and/or the capacity of resident microbiota to produce butyrate had an impact on host susceptibility to infection and disease [19]. Accumulating evidence demonstrates the involvement of the Western diet in gut microbiota shifts that enhance susceptibility to enteric infections in mice, but to date, the effect of the Western diet on EHEC colonization as mediated by changes in gut microbes from humans has yet to be investigated.

Due to obvious ethical restrictions, the impact of the Western diet on EHEC colonization cannot be directly assessed in humans. An alternative to *in vivo* assays is the use of *in vitro* models simulating human digestion with the associated resident intestinal microbes. Different *in vitro* models associating dynamism and gut regionalization have already been developed and validated according to *in vivo* data from humans [20,21,22,23,24]. In particular, the Artificial COLon (ARCOL), which reproduces the main nutritional, physicochemical, and microbial conditions of the human adult large intestine, has been used to investigate EHEC O157:H7 colonization and virulence [7,8]. A recent optimization of this model (M-ARCOL) led to the mucosal configuration, where specific microenvironments colonized with lumen- and mucus-associated microbiota are distinguished [25,26,27,28]. In this context, the aim of the present study was to investigate the effects of a Western diet versus a Standard diet on the colonization of EHEC O157:H7 reference strain EDL933, as mediated by changes in the human colonic microbiota composition and metabolic functions, using the M-ARCOL model. This was performed after an initial selection of four out of nine donors, tested using a simplified batch fermentation model, that were then further used for fecal inoculation of the M-ARCOL model.

## 2. Materials and Methods

### 2.1. Bacterial Strains and Culture Conditions

The EHEC O157:H7 reference strain EDL933 used in this study was originally isolated from Michigan ground beef associated to the 1982 outbreak (ATCC 43895). Bacteria were stored in the laboratory at −80 °C in Luria–Bertani (LB) medium containing 20% glycerol. For batch and M-ARCOL experiments, the EHEC strain was grown overnight in LB broth at 37 °C without shaking until reaching stationary phase.

### 2.2. Fecal Sample Collection and Treatment

**Figure 1:**
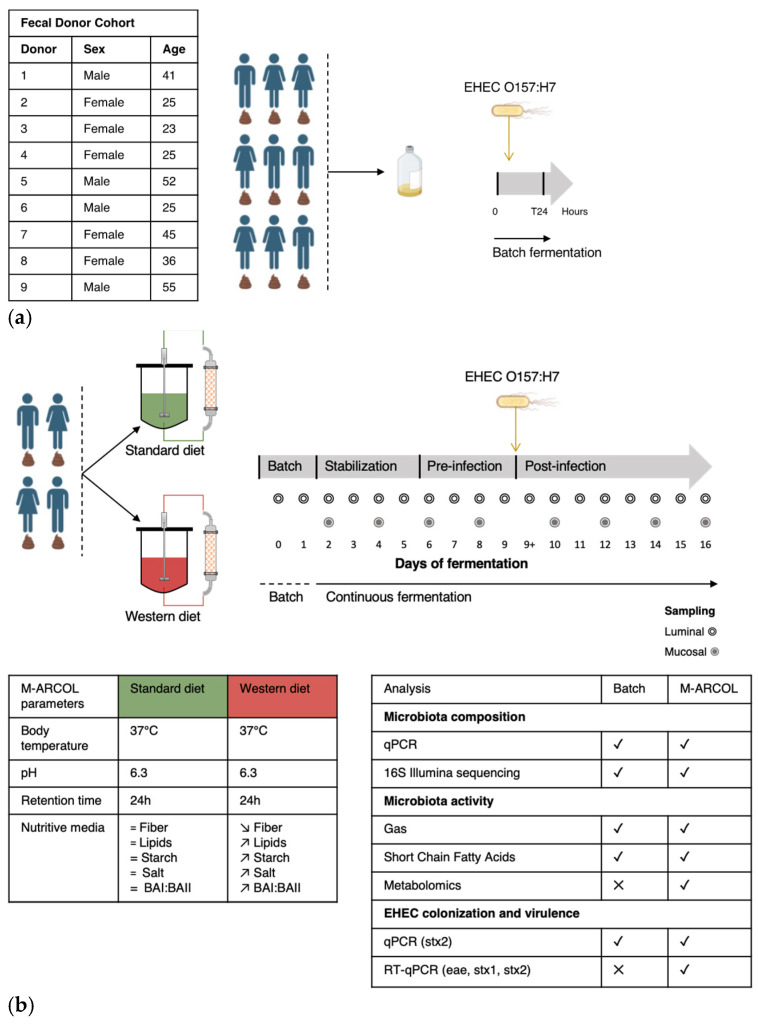
Experimental design of batch experiments and M-ARCOL fermentations. (**a**) An initial pre-screening of donors was performed using a simplified *in vitro* batch fermentation experiment with fecal samples collected from 9 healthy donors (as detailed in the table). Following inoculation with fecal sample, EHEC O157:H7 strain EDL933 was introduced in penicillin flask containing standard nutritive medium. Samples were taken before inoculation (Day 0), immediately after infection (Day 0+) and 24 h after inoculation (Day 1) from the fermentation medium and atmospheric phase. (**b**) Four healthy donors were selected during batch assays to perform M-ARCOL experiments. For each donor (*n* = 4), two bioreactors were inoculated with a human fecal sample and run in parallel, one receiving a Standard diet and the other a Western diet for a total duration of 16 days. After an initial batch amplification (24 h) and a stabilization phase of the gut microbiota, EHEC O157:H7 strain EDL 9333 was introduced in each bioreactor at Day 9. The last three days of the stabilization period (Day 6, 7, 8) were defined as the pre-infection period, while the post-infection period lasted for a further 7 days (from Day 9 to 16). Gas and luminal fermentation media were sampled daily, while mucin–alginate beads were collected every two days for downstream analysis of gut microbiota structure and activity. =: no difference; ↗: higher levels in Western diet versus Standard diet; ↘: lower levels in Western diet versus Standard diet.

Fresh fecal samples were collected anaerobically from 9 healthy adults. These donors were four males (donors 1, 5, 6, 9) and five females (donors 2, 3, 4, 7, 8), ranging in age from 23 to 55 years old (Figure 1), without any history of antibiotic, prebiotic, and probiotic use 3 months prior to the study. Donors all gave written, informed consent to take part in the study. The use of fecal microbiota of human origin was approved by the ethical committee under registration number (MEDIS MICROVITRO 2022-A02066-37). Fecal samples were treated within 6 h following defecation. A 10% inoculum (*w*/*v*) suspension was prepared for each donor’s fecal sample under strict anaerobic conditions (COY Laboratory Products Inc, Grass Lake, MI, USA) by mixing stool in sodium phosphate buffer (pH 6.5) supplemented with 1.9 mM cysteine. The resulting suspension was filtered through a 500 µm sieve in a sterile bottle.

### 2.3. Batch Experiments

Batch experiments were performed in 100 mL penicillin bottles containing nutritive medium (adapted from [25]) and fecal suspension to reach a final volume of 50 mL. The nutritive medium was autoclaved and flushed under CO_2_ flow before use. Batch experimental design and sampling are summarized in Figure 1a. To examine the inter-individual variability of EHEC interactions with the gut microbiota, penicillin bottles were inoculated with fecal samples collected from the 9 healthy individuals. The penicillin flasks were incubated at 37 °C for 24 h under agitation (100 rpm). Aliquots were taken immediately after the start of the incubation (T0), after EHEC infection (T0+), and at 24 h of fermentation from (i) the liquid phase for EHEC quantification by qPCR, microbiome characterization (storage at −80 °C), and SCFA analysis (storage at −20 °C), and (ii) the atmospheric phase for gas analysis.

### 2.4. Fermentations in the M-ARCOL System

M-ARCOL is a one-stage fermentation system operated under semi-continuous conditions, which simulates the nutritional (supply of a nutritive medium reproducing the composition of human ileal effluents), physicochemical (temperature, colonic pH and retention time, anaerobiosis), and microbial conditions (lumen- and mucus-associated microbiota) encountered in the adult colon [25,28]. The model is composed of a main bioreactor (luminal compartment) connected to an external glass compartment (mucosal compartment) containing mucin–alginate beads. The operating parameters (temperature, pH, retention time, volume, stirring speed) as well as the composition of the nutritive medium are given in Figure 1b. In this set of experiments, the composition of ileal effluents supplied as nutritive medium into the main bioreactors was adjusted to contain various sources of carbohydrates, proteins, lipids, minerals, and vitamins to reflect the specificities of a Standard diet [25] or a Western diet [29]. Experimental design and sampling of M-ARCOL experiments are summarized in Figure 1b. Two bioreactors inoculated with fecal stool from one of four healthy donors (*n* = 4) selected in batch experiments (Donor 1, 2, 4, 5) were run in parallel; one bioreactor received the Standard diet, while the other was supplied with the Western diet. All fermentations were run for a total period of 16 days, after a 24 h period of microbial amplification (Figure 1b). The EHEC O157:H7 strain EDL 933 was inoculated at Day 9, defining the pre-infection and post-infection periods. Samples from the main bioreactor were collected daily for EHEC quantification by qPCR and microbiome characterization—referred to as luminal microbiota (storage at −80 °C)—as well as SCFA analysis (storage at −20 °C). Additional samples from the mucin–alginate bead compartment, referred to as mucosal microbiota, were collected every 2 days for gut microbiota characterization. Mucin–alginate beads were washed twice in sterile Phosphate Buffer Saline (PBS) and stored at −80 °C for downstream analyses. In addition, samples were harvested daily from the atmospheric phase to ascertain anaerobic conditions and determine gas composition. The daily volume of gas produced by microbial activity was also measured using a syringe connected to the sampling bag.

### 2.5. DNA Extraction

Genomic DNA was extracted from the luminal samples and mucin–alginate beads using the QIAamp Fast DNA Stool Mini Kit (12830–50, Qiagen, Hilden, Germany) following the manufacturer’s instructions with minor adjustments, as previously described [30,31]. DNA integrity was verified by agarose gel electrophoresis and by Nanodrop 2000 analysis (Thermo Fisher Scientific, Waltham, MA, USA). The DNA quantity was assessed using the Qubit dsDNA Broad Range Assay Kit (Q32851, Invitrogen, Carlsbad, CA, USA) with a Qubit 3.0 Fluorometer (Invitrogen, Carlsbad, CA, USA). Samples were stored at −20 °C before analysis (qPCR and microbiome characterization).

### 2.6. Quantitative PCR

Total bacteria and EHEC kinetics (using *stx2* gene) were quantified in the M-ARCOL by quantitative PCR (qPCR). Primers for total bacteria and EHEC, as well as hybridization temperatures, are listed in Supplementary Table S1. qPCR analysis was performed on a Biorad CFX96TM Real-Time System (Bio-Rad Laboratories, Hercules, CA, USA) using Takyon Low ROX SYBR 2X MasterMix blue dTTP kit (Eurogentec, Serain, Belgium). Each reaction was run in duplicate in a final volume of 10 μL, prepared as previously described in Deschamps et al. [25]. The amplification conditions consisted of 1 cycle at 95 °C for 5 min, followed by 40 cycles of 95 °C for 30 s, annealing temperatures for 30 s, and 72 °C for 30 s. A melting step was added to ensure primer specificity. Standard curves were generated from 10-fold dilutions of bacterial DNA (isolated from reference strains) and allowed the calculation of DNA concentrations from the extracted samples. Results for EHEC colonization are expressed as survival percentage relative to initial inoculum [8].

### 2.7. 16S Metabarcoding Analysis of Gut Microbiota Composition

The bacterial V3-V4 regions of 16S ribosomal DNA (rDNA) were amplified with primers V3_F357_N and V4_R805 (Supplementary Table S1). Amplicons were generated using a Fluidigm Access Array followed by high-throughput sequencing using the Illumina MiSeq system (Illumina, San Diego, CA, USA) performed at the Carver Biotechnology Center of the University of Illinois Urbana-Champaign (Urbana, IL, USA). Bioinformatic analysis was performed using R software version 4.1.2 (1 November 2021) and the DADA2 pipeline [32]. Demultiplexed raw sequence data in fastq format were quality-filtered; reads with N bases were eliminated and reads under 50 bp length were removed. Decontamination steps were carried out to filter out sequences corresponding to PhiX DNA, used as a spikein control for MiSeq runs. Filtered sequences were dereplicated and cleaned for chimeras. Remaining sequences were dereplicated and amplicon variant sequences (ASVs) were inferred using the dada function from the dada2 package. A table with ASV counts for each sample and representative sequences of ASVs in fasta format were generated. Taxonomic affiliation of all ASVs were performed with the assignTaxonomy function using the SILVA database release 138.1 (80% bootstrap cut-off) as a reference [33]. A phylogenetic tree was constructed based on ASV representative sequences using functions from the phangorn package [34] and relative bacterial abundance was plotted at the phylum, family, and genus levels. For the α- and β-diversity analysis, unweighted and weighted Unifrac distance matrices were generated, and a redundancy analysis (RDA) ordination was used for visualization.

### 2.8. Gas Composition Analysis

Gas samples collected during batch and M-ARCOL fermentations were analyzed in a 490 micro-gas chromatograph (Agilent Technologies, Santa Clara, CA, USA) coupled with a Thermal Conductivity Detector (TCD). Two series columns, Molecular Sieve 5A and PoraPlot U, were used. Gas composition was determined using calibration curves made from ambient air (78.09% N_2_, 20.95% O_2_, 0.04% CO_2_) and three gas mixtures, A (5% CO_2_, 5% H_2_, 90% N_2_), B (19.98% CO_2_, 80.02% H_2_), and C (19.89% CO_2_, 19.88% CH_4_, 20% H_2_, 40.23% N_2_). Technical replicates were performed for each sample and results were expressed as relative percentages [26].

### 2.9. Short-Chain Fatty Acid Analysis

A total of 2 mL of fermentation samples collected at T0 and T24 h in batch experiments and obtained at different timepoints in M-ARCOL was centrifuged (14,000× *g*, 15 min, 4 °C) and 900 µL of supernatant was diluted at 1/10 in 0.04 M H_2_SO_4_ mobile phase, vortexed, and filtered (pore size 0.22 μm). These supernatants were used to determine the concentration of the three major SCFAs (acetate, propionate, and butyrate) using High-Performance Liquid Chromatography (HPLC) (Elite LaChrom, Merck KGaA, Darmstadt, Germany) coupled to a diode-array detection (DAD) diode. Results were expressed as mM and relative percentages.

### 2.10. Metabolomic Analysis

Samples for metabolomics analysis were taken from the luminal phase of M-ARCOL bioreactors prior to EHEC infection (Days 8 and 9) and in the post-infection period (Days 10, 12, 14, 16). Four analytical methods were used to quantify different metabolites. Bile acids and SCFAs were quantified with targeted methods using liquid chromatography and gas chromatography, coupled to tandem mass spectrometry (LC-MS/MS and GC-MS/MS, respectively), using multiple reaction monitoring (MRM) optimized for each metabolite. Untargeted polar metabolite profiling was performed using GC-MS using a quadrupole time-of-flight (qTOF) mass spectrometer configured in full-scan mode. Targeted metabolomics data were processed with Agilent MassHunter Quantitative Analysis (version 10.1). Quantification was carried out with a calibration curve prepared with an analytical standard for each bile acid and SCFA. Internal standard correction was used. Untargeted GC-MS data were acquired to analyze metabolites such as carbohydrates, amino acids, hydroxyl acids, and free fatty acids and processed using the R package eRah [35]. Metabolites were annotated via spectral and retention index matching to the data from Golm Metabolome Database (GMD). Additional details on metabolite extraction, acquisition methods, and data analysis are included in Supplementary Materials and Appendix A.

### 2.11. Statistical Analysis

Statistical analyses on gut microbiota activity (gas and SCFA) were processed using GraphPad Prism software version 9 (GraphPad Software, San Diego, CA, USA). Data normal distribution was verified by combining Anderson–Darling, D’Agostino–Pearson, Shapiro–Wilk, and Kolmogorov–Smirnov tests. Then, appropriate statistical analysis was applied (either one-way ANOVA, *t*-test, or Mann–Whitney tests). A *p*-value < 0.05 was considered statistically significant. Metabarcoding analysis of gut microbiota, including α-diversity indexes (number of observed ASVs and Shannon index), was carried out using the R software (version 4.1.2). Principal coordinate analysis (PCoA) was performed followed by Non-Metric Multidimensional Scaling (NMDS), highlighting important donor effects. Constraint Redundancy Analysis (RDA) was then performed, with time, donor, diet, and infection as variables of the model and with the removal of the donor effect. Bray–Curtis distances were used for each analysis and significance between groups was assessed with 95% confidence ellipses. Differential analyses (DESeq2, metagenomeSeq, metacoder) were performed using rANOMALY package [36]. The metabolomics statistical analysis was performed in R (version 4.2.0). R package vegan (version 2.6-4) was used to perform the RDA analysis, while ggpubr (version 0.6.0) was used for making the boxplots. Statistical significance was calculated using the Wilcoxon test unless otherwise specified.

## 3. Results

### 3.1. Selection of Donors from Batch Experiments for M-ARCOL Assays

**Figure 2:**
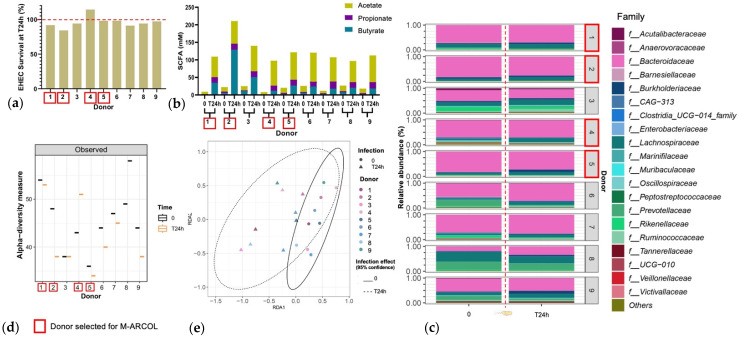
Selection of donors using batch fecal experiments. Penicillin bottles containing nutritive medium were inoculated with feces from 9 healthy donors, and then challenged with EHEC O157:H7 strain EDL933, and samples were collected before (T0) and 24 h after infection (T24h). (**a**) qPCR quantification of EHEC survival, expressed as percentages of initial infection. (**b**) Concentrations of the three main SCFAs in the fermentation medium expressed in mM. (**c**) Relative percentages of the 20 main bacterial populations analyzed in the fermentation medium by 16S metabarcoding at the family level. (**d**) Bacterial α-diversity determined at the ASV level. (**e**) Bacterial β-diversity represented by a distance-based redundancy analysis (RDA) using the Bray–Curtis matrix, excluding the donor variable. Red squares indicate the four donors that have been selected for further M-ARCOL experiments.

An initial pre-screening of donors was performed using a simplified *in vitro* batch fermentation experiment with fecal samples from nine healthy donors, to select the four to be included in the M-ARCOL experiments. EHEC quantification by qPCR revealed inter-individual variations in colonization percentages at 24 h post-inoculation (Figure 2a). Donors 1, 2, 3, 7, and 8 showed EHEC survival lower than 100%, the largest decrease being found with donor 2 (84% survival at 24 h). Donor 4 was the only one to show a surge in EHEC survival (114% survival at 24 h). The other donors (donors 5, 6, and 9) retained a high level of EHEC, at almost 100% T24h post-infection. For all donors, SCFA concentrations were higher at T24h post-inoculation compared to the initial levels (Figure 2b). Inter-individual variability in SFCA production was also evidenced between the donors, ranging from 98 mM for donor 4 to 211 mM for donor 2. This high level in donor 2 was explained by a unique spike in butyrate production. Observed alpha-diversity measure also showed variations between the individual donors (Figure 2c). Donor 1 retained a high level of alpha-diversity with a slight reduction at T24h, and donor 5 retained a low level of alpha-diversity with a slight reduction at T24h. Donor 2 had a clear decrease in alpha-diversity at T24h, whereas donor 4 showed opposite trends. Beta-diversity also shifted following EHEC infection with a clustering occurring with all donors at T24h (Figure 2d). The fecal microbiota of the nine donors used for batch experiment inoculation was characterized by 16S metabarcoding and showed that the bacterial profiles are highly donor-dependent, particularly at the family level (Figure 2e). An increase in *Lachnospiraceae* was evidenced in donors 1, 2, 4, and 5 at T24h following EHEC infection. An increase in *Burkholderiaceae* can be evidenced in donors 1 and 5, but a decrease is observed in donor 2. Four donors were selected out of nine donors profiled via batch assay, namely donor 1 (male, 41 years old), donor 2 (female, 25 years old), donor 4 (female, 25 years old), and donor 5 (male, 52 years old), taking into account the interindividual variability in all those analyses, but also keeping a gender and age balance, with two males and two female donors, ranging in age from 25 to 52 years old, to be used in the M-ARCOL experiments.

### 3.2. Effect of Western Diet on EHEC Colonization in M-ARCOL

**Figure 3:**
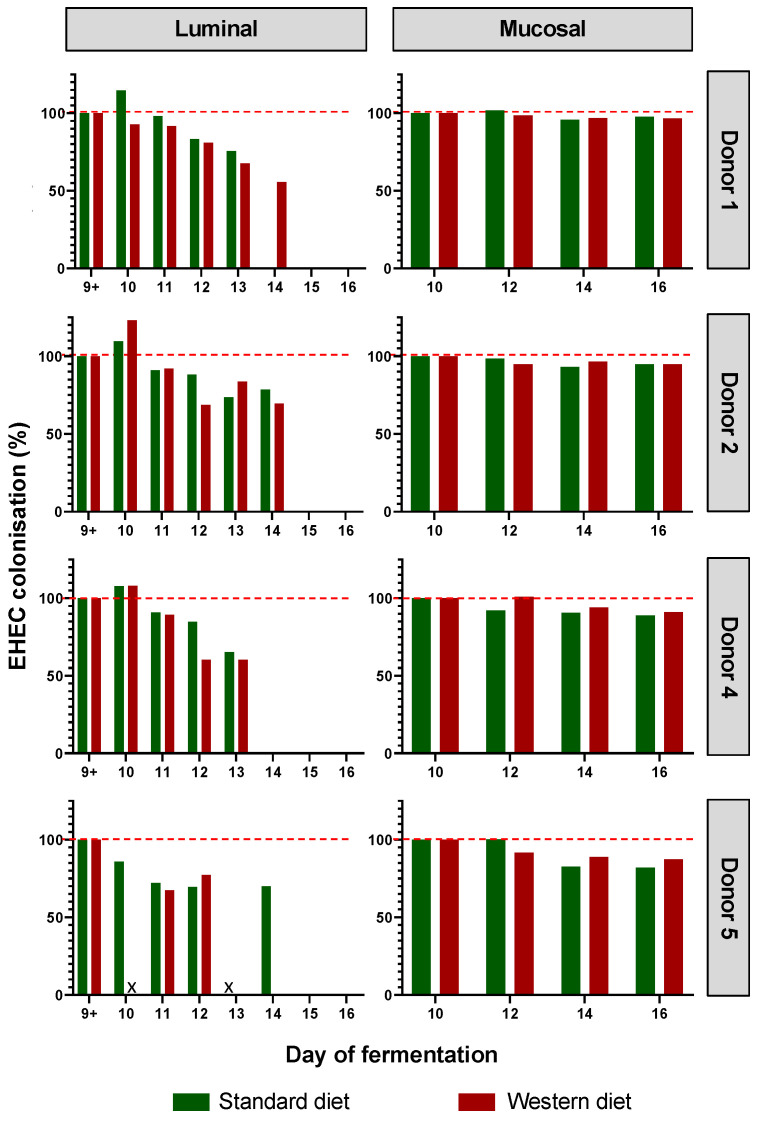
Impact of diet on EHEC colonization in the M-ARCOL. Fermentations were run in the M-ARCOL model; every two bioreactors were inoculated with a fecal sample from one of 4 healthy donors (*n* = 4) supplied with either Standard or Western diet, and challenged on Day 9 with EHEC O157:H7 strain EDL933. Samples were regularly collected after infection in both the luminal and mucosal phases of the *in vitro* colon model. EHEC colonization was followed by qPCR analysis and expressed as percentages of initial infection rate.

EHEC colonization was progressively reduced over the course of fermentation for each donor in the luminal phase of M-ARCOL, while EHEC colonization was maintained close to 100% in the mucosal phase with both the Standard and Western diets (Figure 3). Initial EHEC amounts were maintained at Day 16 in the mucin beads for all donors, while the pathogen had disappeared from the luminal compartment, showing the clear impact of colonic microenvironment on EHEC ability to colonize. The kinetics of EHEC colonization in the luminal phase were subjected to inter-individual variability, with levels decreasing more rapidly in donor 4, compared to donors 1, 2, and 5, where EHEC was still detectable by Day 14. Interestingly, the effect of diet on EHEC colonization was donor-dependent, and was mostly observed in the luminal phase. Over the course of fermentations, similar percentages of EHEC colonization with a Standard diet and a Western diet were observed for donors 2 and 5. On the contrary, in donor 1, the Western diet sustained EHEC colonization until Day 14 of fermentation (70%) compared to an undetectable level with a Standard diet, while opposite trends were observed in donor 5.

### 3.3. Effect of Western Diet on Gut Microbiota Composition in Pre- and Post-Infection with EHEC

**Figure 4:**
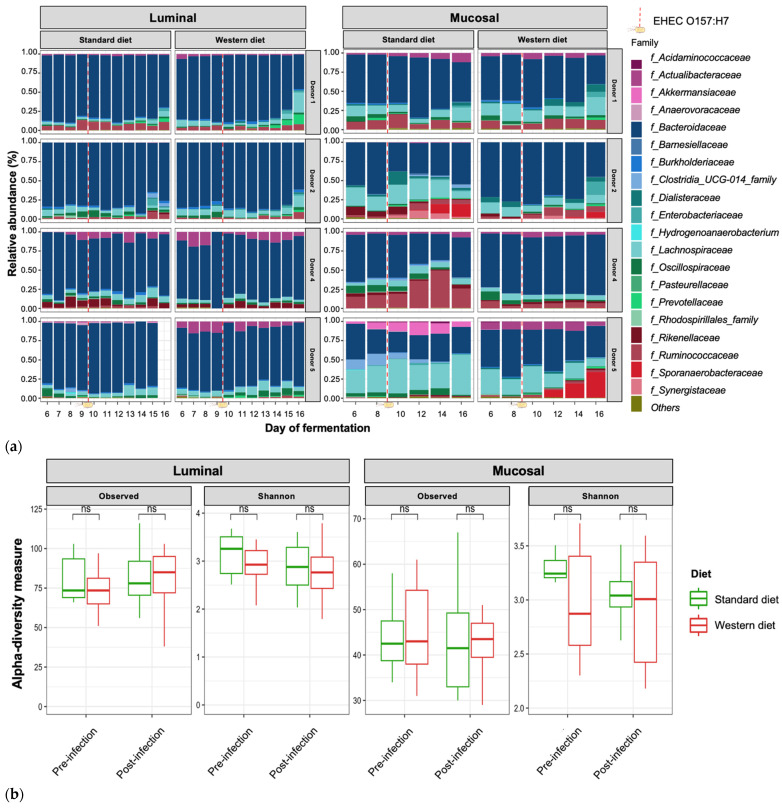

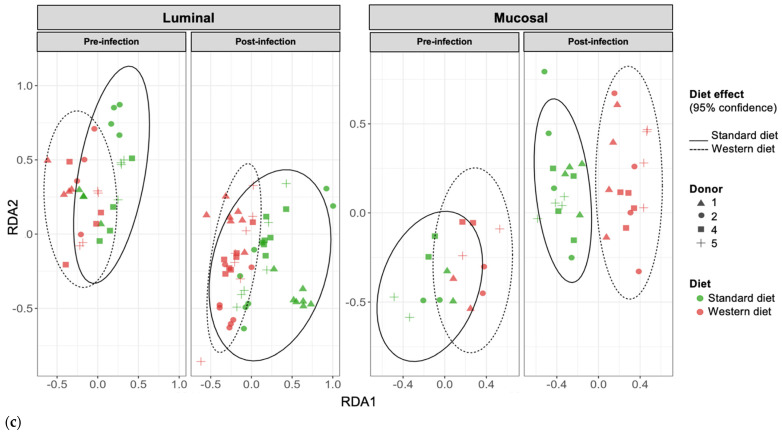
Effect of diet on gut microbiota composition and bacterial diversity in the M-ARCOL challenged by EHEC infection. Fermentations were run in the M-ARCOL model; every two bioreactors were inoculated with a fecal sample from one of four healthy donors (*n* = 4), supplied with either Standard or Western diet, and challenged on Day 9 with EHEC O157:H7 strain EDL933 (dashed line). Samples were regularly collected in the pre- and post-infection periods in both the luminal and mucosal phases of the *in vitro* colon model and gut microbiota composition analyzed by 16S Metabarcoding. (**a**) Relative abundance of the 20 main bacterial populations at the family level. (**b**) Mean bacterial α-diversity expressed as number of observed ASVs and Shannon index (*n* = 4). (**c**) β-diversity represented by a distance-based redundancy analysis (RDA) using the Bray–Curtis matrix, excluding donor effect. Significance between groups was assessed (*p* < 0.05) with 95% confidence ellipses (ns = non-significant).

**Figure 5:**
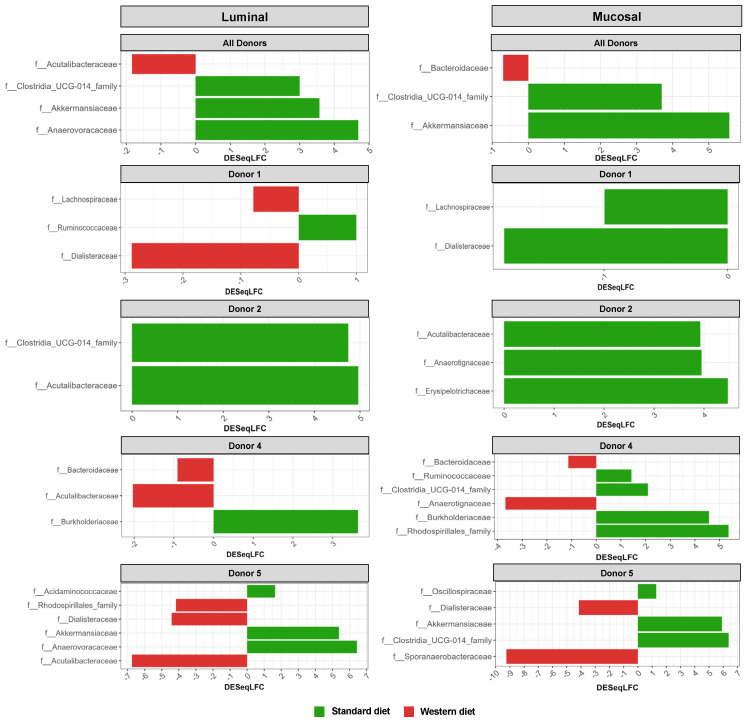
Differential analysis of Standard and Western diets’ effect on bacterial populations in the luminal and mucosal environments of the M-ARCOL. Fermentations were run in the M-ARCOL model; every two bioreactors were inoculated with a fecal sample from one of four healthy donors (*n* = 4), supplied with either Standard or Western diet, and challenged on Day 9 with EHEC O157:H7 strain EDL933. Samples were regularly collected in both the luminal and mucosal phases of the *in vitro* colon model and microbiota composition analyzed by 16S Metabarcoding. Differential analyses were performed with three different methods (DeSeq2, Metacoder, and MetagenomeSeq R-analysis). Red and green color codes indicate the families significantly more abundant in the Western and Standard diets, respectively, for all donors pooled or for each individual donor, through the course of fermentation. Presented families are differentially more abundant in at least one method.

In the pre-infection period, the bacterial abundance profiles at the family (Figure 4a), phylum, and genus levels (Supplementary Figure S1a,b) clearly indicated that the inter-individual variability observed in the initial donor stools and in batch experiments was retained in the *in vitro* model, both in the luminal and mucosal phases. The impact of the diet on bacterial profiles was obvious and donor-dependent. When all donors were pooled, differential analysis highlighted significantly higher abundances of *Akkermansiaceae* and *Clostridia*-UCG014 in both the luminal and mucosal environments (Figure 5) in the context of the Standard diet. At the individual level, a decrease in *Ruminococcaceae* was observed with the Western diet in donors 1, 2, and 4 in the luminal and/or mucosal phases (Figure 4a), significant in donors 1 and 4 (Figure 5). A drop in *Akkermansiaceae* with a Western diet relative to a Standard diet was also noticed in donor 5, especially in the mucosal compartment (Figure 4a), and supported by differential analysis (Figure 5). On the contrary, *Enterobacteriaceae* seemed to increase in donors 1 and 2 under the Western diet condition in the mucosal phase. Donors 1 and 5, who showed opposite trends for diet impact on EHEC colonization, are characterized by different changes in bacterial profiles. An increase in *Prevotellaceae* and *Lachnospiraceae* was observed in donor 1 under the Western diet condition, while for donor 5, a decrease in *Akkermanciaceae* together with an increase in *Acutalibacteraceae* were observed. Donor 2 was the only one to exhibit a significant increase in *Acutalibacteriaceae* under the Standard diet condition in both the luminal and mucosal compartments (Figure 5). Furthermore, no clear shift in bacterial abundance in the post-infection period can be attributed to EHEC infection, whatever the donor or the diet (Figure 4a). Differential analysis revealed that *Enterobacteriaceae* was, as expected, the main family increased in the post-infection phase in the luminal compartment (Supplementary Figure S2). In donors 2 and 5, *Sporanaerobacteriaceae* was also significantly more abundant in the post-infection period, but in the mucosal phase only. Interestingly, the luminal samples were characterized by a clear dominance of *Bacteroidaceae* in all donors and diet conditions, whereas the mucosal samples displayed more variations in main bacterial populations. Regarding α-diversity, observed ASVs and Shannon indexes were not significantly impacted by the diet or infection (Figure 4b). However, the Shannon index tended to be lower in both the luminal and mucosal compartments with the Western diet in the pre-infection period. At the individual level, α-diversity was also not impacted by the Western diet. Donor effect had the stronger impact on luminal and mucosal β-diversity (Supplementary Figure S1c). An RDA analysis excluding this donor effect revealed that the diet had a significant impact (95% confidence) on microbial β-diversity (Figure 4c). The impact of the diet was more striking in the mucosal phase of the colonic environment, mainly during the post-infection period with two clear clusters.

### 3.4. Effect of Western Diet on Gut Microbiota Activity in Pre- and Post-Infection with EHEC

**Figure 6:**
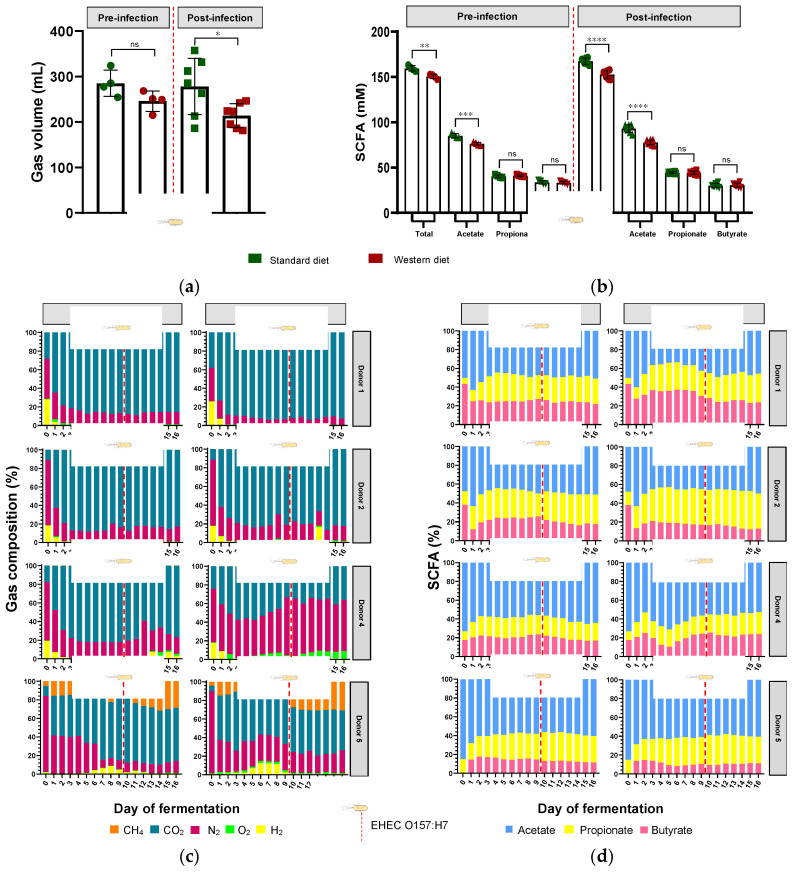
Effect of diet on gut microbiota activities in the M-ARCOL challenged by EHEC infection. Fermentations were run in the M-ARCOL model; every two bioreactors were inoculated with a fecal sample from one of four healthy donors (*n* = 4), supplied with either Standard or Western diet, and challenged on Day 9 with EHEC O157:H7 strain EDL933 (dashed line). Samples were regularly collected in the pre- and post-infection periods in the luminal medium and atmospheric phase of bioreactors. (**a**) Total gas production (mean ± SEM in mL, *n* = 4). (**b**) Total SCFA concentrations (mean ± SEM in mM, *n* = 4). (**c**) Gas composition in percentages for each donor throughout fermentation. (**d**) Short-chain fatty acid (SCFA) composition in relative percentages for each donor throughout fermentation. Significance between groups was assessed by unpaired *t*-test (* *p* < 0.05, ** *p* < 0.01, *** *p* < 0.001, and **** *p* < 0.0001, ns = non-significant). CH_4_: methane, CO_2_: carbon dioxide, H_2_: dihydrogen, N_2_: nitrogen, O_2_: dioxygen.

In parallel with gut microbiota composition, the impact of diet in pre- and post-infection by EHEC O157:H7 was also monitored for gut microbial activities by measuring fermentation gas and SCFAs daily. There was no significant impact of diet on overall atmospheric gas volume in the pre-infection period, when all donors were pooled (Figure 6a); however, gas volume significantly decreased with the Western diet in the post-infection period (*p* < 0.05). Inter-individual variability was visible in the gas composition profiles of each donor (Figure 6c). Of interest, methane was only found in donor 5, suggesting the presence of methanogenic microbes in the microbiome, while donors 1 and 2 exhibited similar gas profiles. Furthermore, the impact of diet on gas composition profiles was donor-dependent, with almost no effect on donor 1 (except a slight increase in CO_2_ in the pre- and post-infection periods) and donor 2. In donor 4, the Western diet led to a rise in O_2_ production, associated with a decrease in CO_2_ level, in both the pre- and post-infection periods. In donor 5, the Western diet was associated with a decrease in CH_4_ before EHEC infection, while CO_2_ and H_2_ were reduced in the post-infection period. EHEC infection had no effect on gas profiles, except in donor 5, where CH_4_ was higher and H_2_ lower in the post-infection period compared to the pre-infection period, most probably due to a time effect rather than a real impact of the pathogen.

When all donors were pooled, the Western diet significantly reduced the total production of SCFAs in both the pre-infection (151 versus 159 mM, *p* < 0.01) and post-infection (153 versus 167 mM, *p* < 0.0001) periods (Figure 6b). This was exclusively due to a change in acetate production, since the Western diet significantly reduced acetate levels in both the pre-infection (76 versus 85 mM, *p* < 0.001) and post-infection (78 versus 93 mM, *p* < 0.0001) periods. Interindividual variability was also observed in SCFA profiles and the impact of diet on relative percentages of acetate, propionate, and butyrate (Figure 6d). In donor 1, Western diet tended to increase butyrate production and decrease acetate levels, while in donor 2, butyrate decreased when propionate increased. Interestingly, donor 1 had the highest percentages of butyrate, while donor 5 exhibited the lowest ones under Western diet conditions, and similar differences were obtained when results were expressed in concentrations (Figure 6d and Figure S3). Opposite trends were evidenced for acetate in these two donors (Figure 6d and Figure S3). In addition, no impact of EHEC infection on SCFA percentages was noticed, irrespective of the donor.

### 3.5. Effect of Western Diet on Metabolomic Profiles Pre- and Post-Infection with EHEC

**Figure 7:**
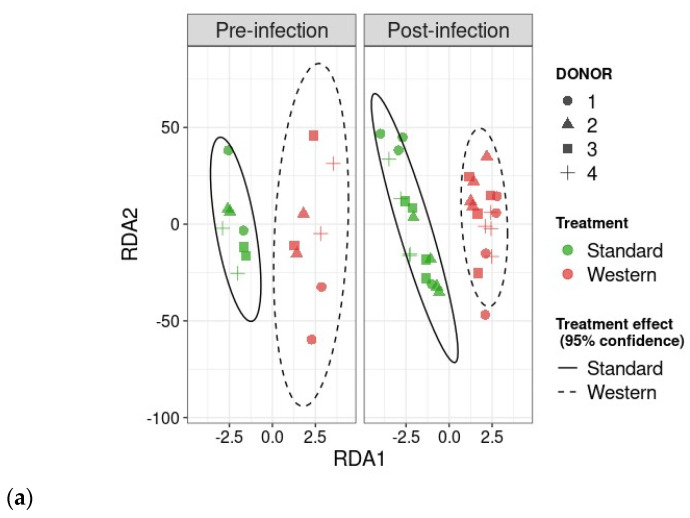

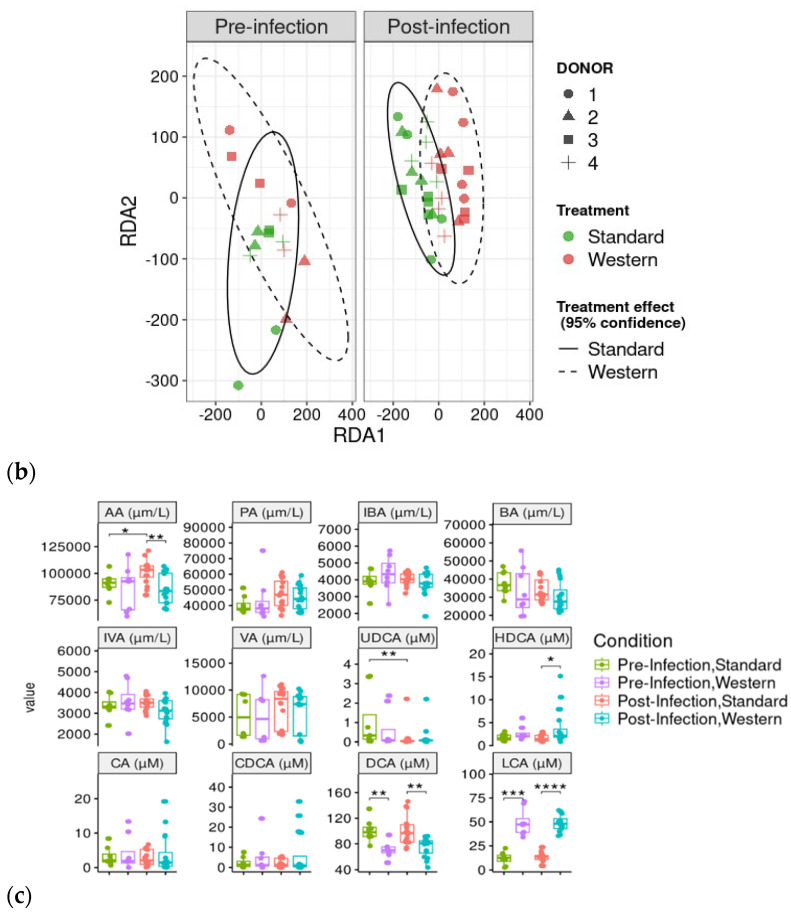
Effect of diet and infection periods on SCFA and bile acid profiles. Fermentations were run in the M-ARCOL model; every two bioreactors were inoculated with a fecal sample from one of four healthy donors (*n* = 4), supplied with either Standard or Western diet, and challenged on Day 9 with EHEC O157:H7 strain EDL933. Samples for metabolomic analysis were taken from the luminal phase of M-ARCOL during the pre-infection (Days 8 and 9) and post-infection (Days 10, 12, 14, 16) periods. RDA analysis of bile acids (**a**) and SCFAs (**b**) in pre-infection and post-infection periods for both Standard and Western diet using the Bray–Curtis matrix excluding donor effect. Significance between groups was assessed by 95% confidence ellipses. (**c**) Quantification of bile acids and SCFAs with targeted methods using liquid and gas chromatography coupled to tandem mass spectrometry, respectively. Results are expressed as mean values (*n* = 4). Significant differences between groups (Wilcoxon test) are indicated (* *p* < 0.05, ** *p* < 0.01, *** *p* < 0.001, and **** *p* < 0.0001). SCFA abbreviations. Acetic acid: AA; propionic acid: PA; isobutyric acid: IBA; butyric acid: BA; IVA: isovaleric acid; VA: valeric acid. Bile acid abbreviations. Cholic acid: CA; chenodeoxycholic acid: CDCA; deoxycholic acid: DCA; hyodeoxycholic acid: HDCA; lithocholic acid: LCA; ursodeoxycholic acid: UDCA.

**Figure 8:**
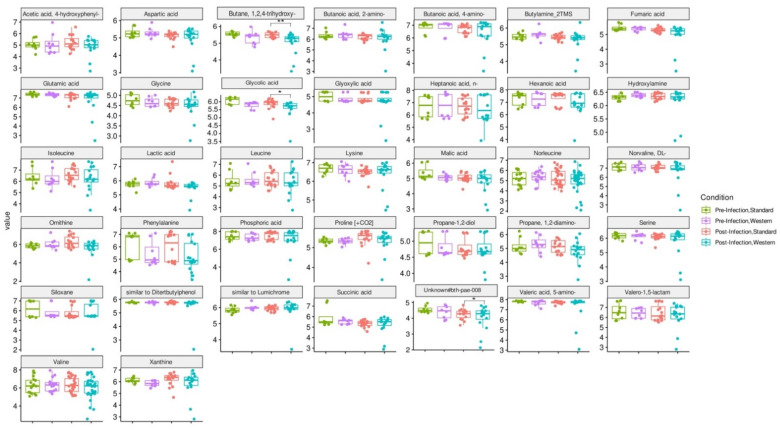
Effect of diet and infection periods on metabolic profiles as assessed by non-targeted metabolomics. Fermentations were run in the M-ARCOL model; every two bioreactors were inoculated with a fecal sample from one of four healthy donors (*n* = 4), supplied with either Standard or Western diet, and challenged on Day 9 with EHEC O157:H7 strain EDL933. Samples for metabolomic analysis were taken from the luminal phase of M-ARCOL during the pre-infection (Days 8 and 9) and post-infection (Days 10, 12, 14, 16) periods. Untargeted GC-MS was used to analyze derivatized compounds by GC-qTOF and 39 metabolites with RI error < 1% and spectral similarity > 90% were manually identified using GMD. Results are expressed as mean values (*n* = 4). Significant differences between groups (Wilcoxon test) are indicated (* *p* < 0.05 and ** *p* < 0.01).

RDA analysis highlighted that the Western diet had a clear impact on bile acid profiles, both in the pre- and post-infection periods (Figure 7a). The effect of the Western diet was less distinct, but still visible on SCFA profiles (Figure 7b). In the post-infection period, mainly when subjected to a Western diet, bile acids and SCFAs were less dispersed compared to the pre-infection period. When all donors are pooled, targeted analysis of SCFAs showed a significant (*p* < 0.05) increase in acetic acid in the post- versus pre-infection period under the Standard diet, as well as a drop in acetic acid in the post-infection period with the Western diet compared to the Standard diet (*p* < 0.01). Those results can be linked to those previously obtained in Figure 6b. Regarding bile acids, in the post-infection period, Western diet was associated with significant increases in hyodeoxycholic acid (HDCA) (*p* < 0.05) and lithocholic acid (LCA) (*p* < 0.001), but a decrease in deoxycholic acid (*p* < 0.01). Of interest is that similar trends were also observed in the pre-infection period. The only effect of EHEC infection led to a significant decrease in ursodeoxycholic acid (UDCA) when a Standard diet was supplied. Among the 39 metabolites identified by non-targeted metabolomics, none were impacted by EHEC infection, but two metabolites were impacted by the diet in the post-infection period, in particular butane—1,2,4 trihydroxy (** *p* < 0.01) and glycolic acid (* *p* < 0.05) (Figure 8).

## 4. Discussion

In recent decades, dietary habits in Western societies have shifted towards a Western-style diet, which is characterized by increased intakes of foods that are low in fiber but high in fat, starch, salt, and sugar [29]. Based on these dietary changes, we adapted the M-ARCOL model to replicate the human colonic physicochemical and nutritional environment associated with a Western diet. This model was used for the first time to investigate the impact of a Western-like diet on food-borne EHEC O157:H7 strain EDL933 survival in the presence of a complex human-derived microbiome. This study is particularly significant, since investigations cannot be performed in human clinical trials due to obvious ethical restrictions with pathogenic infection. The human gut microbiota can protect against invading pathogens through the phenomenon of “colonization resistance”, a multifactorial process including direct inhibition of pathogenic colonization through inhibitory molecules, competition for nutrients, or activation of the host immune system [37]. The Western diet, which is low in microbiota-accessible carbohydrates, has been associated with a reduction in fecal microbial diversity and even a loss of certain bacterial species in humans [38]. Similarly, in rodent models of EHEC infection, a low-fiber Western-style diet altered gut microbiota composition and was linked to increased susceptibility to the intestinal pathogen *C. rodentium*, with an inability to clear the pathogen [10,39].

Western diet and EHEC colonization. In the present study, we demonstrated that EHEC O157:H7 colonization in the M-ARCOL was dependent on both the donor and diet in the luminal phase of the *in vitro* colonic model, with a progressive elimination of the pathogen over the course of fermentation. Conversely, EHEC was maintained with around 100% survival rate in the mucin beads, highlighting the impact of the colonic microenvironment on the pathogen’s ability to colonize. The inter-individual variability in EHEC colonization has been evidenced in previous *in vitro* studies in human gut models [7,40], which be related to different susceptibilities of humans to the life-threatening complications of EHEC [1,2,3]. In the present study, EHEC O157:H7 strain EDL933 persisted much longer in the M-ARCOL luminal phase compared to previous studies in the same model without the mucus phase [7,40]. These data reinforce the importance of integrating the mucus compartment, especially when investigating enteric pathogen pathophysiology. The addition of the mucosal compartment most likely provides nutrients that EHEC can utilize in the luminal phase after degradation, as well as a physical niche for the pathogen to adhere to. Similarly, *in vivo*, the mucosal layer of the intestinal epithelium may offer an important niche for EHEC colonization [9,41,42,43,44]. EHEC has an advantage over commensal *E. coli*, as it produces proteins that can release mucin sugars, such as mucin-degrading zinc metalloprotease StcE, which allows competitive consumption of this nutrient source and promotes penetration of the intestinal mucosal layers, and adhesion and colonization of EHEC [3,45,46,47]. In addition, EHEC O157:H7 has the genetic repertoire to utilize all sugars present in mucins; in particular, fucose, galactose, and GlcNAc consumption appears to be the most important sugars for effective colonization of EHEC strain EDL933 in mice [3,44]. Neumann et al. (2021) demonstrated in mice that the absence of dietary fiber intake resulted in microbiome-mediated erosion of the colonic mucus barrier and increased markers of mucosal barrier integrity disruption, making individuals more susceptible to infection by *C. rodentium* [39]. In our *in vitro* study, we did not evidence any effect of the Western diet on mucin bead erosion, as indicated by the weight of mucin bead samples extracted from Western diet mucosal compartment compared to the Standard diet mucosal compartment and associated EHEC colonization in the mucosal phase.

Impact of diet and EHEC infection on human gut microbiota structure. In the luminal compartment of M-ARCOL, the Western diet sustained EHEC colonization in donor 1, while opposite trends were observed in donor 5, and no effect was evidenced in donors 2 and 4. The Western diet showed an obvious donor-dependent impact on gut bacterial profiles, but no clear link could be made between those shifts in gut microbiome and EHEC ability to survive *in vitro*. However, donors 1 and 5 were characterized by different changes in their bacterial profiles in the luminal phase associated with the Western diet, i.e., an increase in *Prevotellaceae* and *Lachnospiraceae* abundances in donor 1, and a drop in *Akkermanciaceae* in donor 5. The Western diet appears to cause a loss in microbial markers of health, such as *Akkermansiaceae*, *Rikenellaceae*, and *Ruminococcaceae*, and an increase in markers of dysbiosis, such as *Prevotella* and *Proteobacteria*, as demonstrated in humans, based on fecal stool analysis [48]. In the M-ARCOL model, the Western diet led to microbial perturbations, but not in all donors. For instance, with the Western diet, *Ruminococcaceae* abundance decreased in donors 1, 2, and 4, *Akkermansiaceae* in donor 5, and *Rikenellaceae* in donors 2 and 4. The depletion of *Akkermansiaceae* (*Verrucomicrobia* phylum) has been linked to diets low in fiber and high in fat or salt in mice or humans [49,50]. *Akkermansiaceae* is known as an “anti-obesity” microbe, which inversely correlates with the onset of inflammation and metabolic disorders in both mice and humans [51,52]. *Rikenellaceae* (*Bacteroidetes* phylum) tends to be present in the gut microbiota of individuals with good metabolic health, associated with reduced visceral adipose tissue and healthier metabolic profile in the elderly. It has been postulated as an adiposity modulator through the production of the SCFAs acetate and propionate [53,54,55,56]. In humans, a high-fat/low-fiber diet has been associated with a decrease in gut microbial diversity [14], which can further impact susceptibility to pathogen infection. In this study, α-diversity was not reduced when the Western diet conditions were applied to the M-ARCOL model (despite a tendency to diminish with the Shannon index), and prolonged EHEC colonization in donor 1 was not associated with a drop in α-diversity. Therefore, based on our *in vitro* results, we cannot directly link pathogen clearance and loss of gut microbial diversity. This is in accordance with results obtained in mice infected by *C. rodentium* by Neumann and colleagues [39].

Impact of diet and EHEC infection on human gut microbiota functions. Western diet alteration in gut microbiota composition may have a knock-on effect on gut microbiota metabolic activities, leading to changes in SCFA production and bile acid metabolism [11,57]. Deficiency in dietary fiber alters gut microbiota composition and subsequent SCFA production in mice and may contribute to “Western lifestyle” inflammatory diseases and allergies [16,58]. In the M-ARCOL model, when data from all donors were pooled, the Western diet significantly reduced total production of SCFAs, due to a significant decrease in acetate levels in both pre- and post-infection periods. In the human gut microbiota, the predominant acetate-producing bacteria are *Bacteroidetes* as well as *Prevotella* spp., *Bifidobacterium* spp., *Ruminococcus* spp., and *Akkermansia muciniphila* [59]. Therefore, in the *in vitro* colon model, the drop in acetate levels may be linked to the drop in *Bacteriodaceae*, *Ruminoccocaceae*, and *Akkermansiaceae* families. SCFA profiles displayed inter-individual variability and the impact of the diet was also observed at the donor level. The most striking differences were that, under the Western diet conditions, donor 1 exhibited almost double the percentage of butyrate compared to donor 5 together with less acetate, and both donors showed opposite trends regarding the impact of Western diet on EHEC colonization. Although our *in vitro* results did not establish a direct link between butyrate production and EHEC survival, these findings are in line with those obtained in a previous study in mice. Zumbrun et al. demonstrated that a high-fiber diet produced high concentrations of butyrate, which was associated with increased EHEC colonization and subsequent Shiga toxin production [19]. Butyrate upregulates the pathogenicity island locus of enterocyte effacement (LEE) gene expression in EHEC (EHEC O157 Sakai and strain derivatives) culture and promotes adhesion by increasing the expression of virulence genes [60]. Increased butyrate production in donor 1 with Western diet could be associated with a significant increase in *Lachnospiraceae* in the mucosal phase, since this family is known to be a dominate producer of butyrate in the human gut [61,62]. Regarding acetate, there are contradictory results in the literature, which precludes any conclusion on the relation between acetate levels and EHEC colonization in the present study. SCFAs, especially acetate, have been shown to increase *C. rodentium* growth and virulence in mice [10]. However, acetate production had a protective effect against EHEC O157:H7 infection in mice by inhibiting Shiga toxin translocation and improving epithelial cell defense [63,64]. Acetate and propionate alone or in combination also reduced EHEC O157:H7 motility in anaerobic culture [3,65]. Lastly, fermentation gases were also followed as main end-fermentation products. The Western diet was associated with a significant decrease in gas volume in the post-infection phase, most probably associated with the lower amount of fiber found in this diet [66,67]. At the individual level, the most noticeable difference in gas profile was methane production in donor 5 exclusively, and consequently, lower percentages of CO_2_ in donor 5 compared to donor 1. There are no available data in the literature regarding EHEC infection and methanogenic *Archaea*, but our results suggest that this point may deserve more attention, since an *in vitro* study investigating the impact of dietary fiber on the gut microbiota of humans has shown that a high-fiber diet led to an increase in *Archaea* [67]. Bile acids were also followed during M-ARCOL fermentation, since they are impacted by gut microbial activities [57,68]. To simulate a Western-like diet, in addition to nutritional changes, bile acid profiles were also adjusted in the *in vitro* colon model to reflect the impact of a high-fat diet. We therefore increased the primary (cholic and chenodeoxycholic acid)-to-secondary bile acid (deoxycholic acid) ratio in the nutritive medium supplied to bioreactors. This allowed us to reproduce *in vitro* the bile acid reabsorption deficiency that is associated with the overproduction of bile acids when high amounts of lipids are ingested [69,70]. In the post-infection phase, we found that the Western diet led to a significant increase in hyodeoxycholic acid and lithocholic acid, but a decrease in deoxycholic acid. This results from both differences in the nutritive medium between the Standard and Western diets and gut microbial activities inside M-ARCOL, leading to further metabolization of primary into secondary bile acids. Of note *Acutalibacteraceae* was significantly increased with the Western diet in the luminal phase, and this family is known as a bile acid-related microbe, involved in the transformation of bile acids in dairy cows [71]. The only effect of EHEC infection on bile acid profiles observed was a significant decrease in ursodeoxycholic acid with a Standard diet, which cannot yet be explained by the available literature. In the present study, we did not analyze the inter-individual variations in bile acid profiles, so no link could be established between difference in EHEC colonization and changes in bile acids. However, it is known that bile acids can modulate various aspects of EHEC virulence, including motility, adhesion, iron uptake, and Shiga toxin production [3]. In addition, Gadishaw-Lue et al. recently showed that bile salts can serve as an environmental cue for EHEC, enhancing resistance to several key host defense peptides [72].

Limitations and future perspectives. Despite the model’s relevance compared to the physiological situation in humans, M-ARCOL does not reproduce pathogen interactions with host cells, such as EHEC attaching and effacing (A/E) lesions on intestinal epithelia, which are key steps in pathogenesis [3,9,11]. Western diet does tend to tilt gut microbiota balance towards the growth of deleterious bacteria, which can result in inflammation of the intestine, an increase in gut permeability, and release of bacterial endotoxins, activating the inflammatory cascade [73]. In this context, gut inflammation can increase susceptibility to enteric pathogens [74,75]. To better consider these key aspects of EHEC pathogenesis in the context of Western diet, future model developments will include the coupling of M-ARCOL with epithelial and immune intestinal cells of human origin [76]. A combined cellular–colon *in vitro* model could be developed to further investigate the effect of diet, and remediation strategies such as probiotics or prebiotics, on EHEC colonization, virulence, and interactions with host intestinal cells.

## 5. Conclusions

This study provides significant insights into the impact of Western diet on EHEC O157:H7 colonization using a human simulated colonic environment. EHEC survival was dependent on both donor and diet, only in luminal samples. The mucosal compartment retained the pathogen, suggesting a favorable niche environment, perhaps acting as a reservoir. The Western diet had individual dependent effects on bacterial, SCFA, and bile acid profiles. This current study provides a solid foundation in demonstrating the application of a complex *in vitro* model to provide insights into the diet, microbiota, and pathogen interactions in the human gut. 

## Data Availability

The 16S metabarcoding data were deposited and are publicly available in the NCBI Sequence Read Archive (SRA) database with accession number PRJNA1102311.

## Supplementary Materials

The following supporting information can be downloaded at: https://www.mdpi.com/article/10.3390/nu16132046/s1, Figure S1: Effect of diet on microbiota composition in the M-ARCOL challenged with EHEC; Figure S2: Differential analysis of pre- versus post-infection effect on bacterial composition in the luminal and mucosal environments of the M-ARCOL; Figure S3: Effect of diet on individual SCFA production in the M-ARCOL challenged by EHEC infection; Table S1: Primers used for qPCR quantification of EHEC strain and 16S Metabarcoding; Table S2: Details of the optimized MRM transition for each analyte in the bile acids LC MS/MS method; Table S3: Details of the optimized MRM transition for each analyte in the short chain fatty acids GC-MS/MS method.

## Appendix A. Metabolomics Analysis

Supplementary methods for metabolomic analysis (Bile acids Analysis by LC-MS/MS; Short chain fatty acids analysis by GC-MS/MS; Untargeted GC-MS).

Four analytical methods were used to quantify different metabolites. Bile acids and short chain fatty acids (SCFAs) were quantified with targeted methods using liquid chromatography and gas chromatography, coupled to tandem mass spectrometry (LC-MS/MS) and (GC-MS/MS) respectively using multiple reaction monitoring (MRM) optimized for each metabolite. Targeted metabolomics data was processed with Agilent MassHunter Quantitative Analysis (version 10.1). Quantification was done with a calibration curve prepared with an analytical standard for each bile acid. Internal standard correction was used. Untargeted GC-MS data was acquired to analyze metabolites such as carbohydrates, amino acids, hydroxyl acids and free fatty acids and processed using the R package eRah [35]. Metabolites were annotated via spectral and retention index matching to reference data. The metabolomics statistical analysis was performed in R (version 4.2.0). R package vegan (version 2.6-4) was used to perform the RDA analysis while ggpubr (version 0.6.0) was used for making the boxplots. Statistical significance was calculated using the Wilcoxon test unless specified. Additional details on metabolite extraction, acquisition methods and data analysis are included in Supplementary Materials.

### Appendix A.1. Bile Acids Analysis by LC-MS/MS

#### Appendix A.1.1. Sample Preparation

100 mg sample was added to 400 µL of methanol which was subsequently vortexed and centrifuged (at maximum speed for 5 min, 4 °C). The supernatant was collected and 20 µL of internal standard, Cholic Acid-d5 (CA-d5), was added and evaporated in a SpeedVac at 45 °C. Samples were reconstituted with 50 μL of methanol:water (1:1, *v*/*v*) and transferred to glass vials for targeted analysis.

#### Appendix A.1.2. LC-MS/MS

An Agilent UHPLC 1290 Infinity II Series coupled to a QqQ/MS 6490 Series (Agilent Technologies, Santa Clara, CA, USA) was used for the targeted bile acid analysis with Phenomenex Kinetex EVO C18 (150 × 2.1 mm) chromatographic column for metabolite separation. Mobile Phase A was 0.1% ammonium hydroxide and 10 mM ammonium acetate and B was Acetonitrile. The column temperature was set at 27 °C, the injection volume was 2 µL while the flow rate was 0.4 mL/min. The gradient employed was 0–0.08 min, 25% B; 0.08–7.2 min, 25–30% B; 7.2–13.2 min, 30–50% B; 13.2–13.5 min, 50–100% B; 13.5–15.0 min, 100% B; 15.0–15.5 min, 100–25% B; 15.5–17.5 min, 25% B. Data was acquired in negative electrospray ionisation mode (ESI-). The source conditions included 25 psi for nebuliser gas, 200 °C for gas temperature, 14 L/min for gas flow, 250 °C for sheath gas temperature, 11 L/min for sheath gas flow, 3000 V for capillary voltage, and 1500 V for nozzle voltage. Quantitative determination was performed using the multiple reaction monitoring (MRM) mode. The transitions and the retention time for each compound are detailed in Supplementary Table S2.

### Appendix A.2. Short Chain Fatty Acids Analysis by GC-MS/MS

#### Appendix A.2.1. Sample Preparation

Samples were centrifuged at 4 °C and the supernatant was collected and filtered. 10 µL of luminal phase samples were mixed with 10 µL of acidified water (15 % phosphoric acid) and 10 µL of internal standards (AA-LAB at 6000 µmol/L, PA-LAB at 2000 µmol/L, BA-LAB at 1200 µmol/L and IBA-LAB, VA-LAB, IVA-LAB and HA-LAB at 600 µmol/L). Consequently, a liquid-liquid extraction was performed using 1500 µL of MTBE. Samples were vortexed for 10 min following which they were centrifuged at 1500 rpm for 10 min at 4 °C. 50 µL were transferred into a vial with an insert and 50 µL of MTBE were added, to dilute the sample. The vials were centrifuged at 1000 rpm for 30 s at 4 °C and 1.0 µL was injected for GC-MS/MS analysis [77].

#### Appendix A.2.2. GC-MS/MS

An Agilent 7890 GC coupled to QqQ 7000 Series and DB-FFAP (30 m × 0.250 mm × 0.25 µm) analytical column from Agilent Technologies were used for the quantification of SCFAs. Briefly, SCFAs were separated on DB-FFAP chromatographic column (30 m × 0.25 mm × 0.25 μm). The oven temperature was programmed as follows: (i) initial temperature 40 °C, (ii) linearly raised at 12 °C/min to 130 °C (0 min), (iii) then linearly raised at 30 °C/min to 200 °C (0 min) and (iv) in the final step the temperature was ramped at 100 °C/min to 250 °C (4.5 min). The column flow was set at 1.5 mL/min using He as carrier gas. The injector was set at 250 °C and the extract was injected in a splitless mode. The Ionisation was carried out by electronic impart (70 eV) and mass analyser operates on Multi Reaction Monitoring (MRM). The transitions and the retention time for each compound are detailed in the Supplementary Table S3.

### Appendix A.3. Untargeted GC-MS

#### Appendix A.3.1. Sample Preparation

100 µL of sample was mixed with methanol: water (8:2, *v*/*v*). Then, the samples were vortexed and centrifuged (15,000 rpm, 4 °C, 10 min). The supernatant was evaporated to dryness and freeze lyophilizate before compound derivatization (methoxymation and silylation).

#### Appendix A.3.2. GC-MS

The derivatized compounds were analysed by GC-qTOF (model 7200 of Agilent, USA). Chromatographic separation was based on Fiehn Method, using a J&W Scientific HP5-MS 30 m × 0.25 mm i.d., 0.25 µm film capillary column and helium as carrier gas using an oven program from 60 to 325 °C at 10 °C/min. Ionization was done by electronic impact (EI), with electron energy of 70 eV and operated in full Scan mode (50–600 *m*/*z* at 5 spectra/s).

#### Appendix A.3.3. GC-MS Data Processing and Analysis

Vendor specific raw data was converted to mzXML files using Proteowizard. eRah (version 1.1.2) [35] was used for deconvolution, alignment, quantification and metabolite identification. The experimental deconvolved spectra were matched to metabolite reference spectra and retention index (RI) data using the Golm Metabolome Database (GMD). Finally, 39 metabolites with RI error < 1% and spectral similarity > 90% were manually identified using GMD (<1% RI error and >90% of spectral similarity).
