# Supplementary material for: Impact of Western Diet on Enterohemorrhagic Escherichia coli Colonization in the Human In Vitro Mucosal Artificial Colon as Mediated by Gut Microbiota
Source: Nutrients. 2024 Jun 27;16(13):2046. doi: 10.3390/nu16132046 (PMC11243482; doi:10.3390/nu16132046)
Supplement: Supplementary file 1 [file nutrients-16-02046-s001.zip › nutrients-3062169-supplementary.pdf]

Article

Impact of Western Diet on Enterohemorrhagic *Escherichia coli* Colonization in the Human *In Vitro* Mucosal Artificial Colon as Mediated by Gut Microbiota

Deborah O'Sullivan [1], Trisha Arora [2,3,4], Claude Durif [1], Ophélie Uriot [1], Morgane Brun [1], Marc Riu [2], Elisabet Foguet-Romero [2], Iris Samarra [2], Xavier Domingo-Almenara [2,3,4], Cormac G. M. Gahan [5,6,7], Lucie Etienne-Mesmin [1,†] and Stéphanie Blanquet-Diot [1,*,†]

[1] UMR 454 INRAe, Microbiology, Digestive Environment and Health (MEDIS), Université Clermont Auvergne, 28 Place Henri Dunant, F-63000 Clermont-Ferrand, France; deborah.o_sullivan@uca.fr (D.O.); claude.durif@uca.fr (C.D.); uriot.ophelie@hotmail.fr (O.U.); morgane.brun@outlook.fr (M.B.); lucie.etienne-mesmin@uca.fr (L.E.-M.)
[2] Centre for Omics Sciences (COS), Unique Scientific and Technical Infrastructures (ICTS), Eurecat—Technology Centre of Catalonia & Rovira i Virgili University Joint Unit, 43204 Reus, Spain; trisha.arora@eurecat.org (T.A.); marc.riu@eurecat.org (M.R.); iris.samarra@eurecat.org (I.S.); xavier.domingoa@eurecat.org (X.D.-A.)
[3] Department of Electrical, Electronic and Control Engineering (DEEEA), Universitat Rovira i Virgili, 43007 Tarragona, Spain
[4] Computational Metabolomics for Systems Biology Lab, Eurecat-Technology Centre of Catalonia, 08005 Barcelona, Spain
[5] APC Microbiome Ireland, University College Cork, T12 YT20 Cork, Ireland; c.gahan@ucc.ie
[6] School of Microbiology, University College Cork, T12 K8AF Cork, Ireland
[7] School of Pharmacy, University College Cork, T12 K8AF Cork, Ireland
* Correspondence: stephanie.blanquet@uca.fr; Tel.: +33-(0)4-73-17-83-90
† These authors contributed equally to this work.

Supplementary Materials

Table S1. Primers used for qPCR quantification of EHEC strain and 16S Metabarcoding.

Gene	Target	Primer sequence 5′-3′	Reference
qPCR			
16S	Total bacteria	ACTCCTACGGGAGGCAG<br>GTATTACCGCGGCTGCTG	[7]
stx2	Shiga-toxin 2	TTGCTGTGGATATACGAGGGC<br>TCCGTTGTCATGGAAACCG	[7]
Illumina sequencing			
16S	Bacteria	CCTACGGGNGGCWGCAG<br>GACTACHVGGGTATCTAATCC	[76]

Table S2. Details of the optimized MRM transition for each analyte in the bile acids LC MS/MS method.

Bile acid	Retention time (min)	Precursor ion (m/z)	Product ion (m/z)	CE (V)
**CA**	2.001	407.3	343	36
		407.3	345.3	32
**CA d5**	1.982	412.3	348	36
		412.3	350.3	32
**CDCA**	3.928	451.3	391.3	28
		451.3	373,2	44
**DCA**	4.561	391.3	345.1	32
		391.3	343.2	44
**HDCA**	2.169	451.3	391.3	12
		451.3	59	56
**LCA**	8.547	435.3	375.2	16
		435.3	58.8	52
**UDCA**	1.752	451.3	391.3	12
		451.3	59	52

Abbreviations. Cholic acid: CA; Chenodeoxycholic acid: CDCA; Deoxycholic acid: DCA; Hyodeoxycholic acid: HDCA; Lithocholic acid: LCA; Ursodeoxycholic acid: UDCA

Table S3. Details of the optimized MRM transition for each analyte in the short chain fatty acids GC-MS/MS method.

Analyte	Retention time (min)	Quantitative transition (m/z)	Qualitative transition (m/z)	CE (V)
**AA-LAB**	6.82	62→45	62→46	5 /15
**PA-LAB**	7.62	77→58	79→58	5 / 15
**IBA-LAB**	7.87	94→76	94→57	5 / 15
**BA-LAB**	8.43	62→44	75→57	5 / 5
**IVA-LAB**	8.64	63→44	74→46	15 / 5
**VA-LAB**	9.06	63→44	77→58	15 / 5
**HA-LAB**	9.63	63→44	77→58	15 / 5
**AA**	6.82	60→45	60→43	10 / 5
**PA**	7.7	73→55	74→55	5 / 10
**IBA**	7.95	88→73	88→55	10 / 15
**BA**	8.43	60→42	73→55	10 / 5
**IVA**	8.71	60→42	60→45	10 / 10
**VA**	9.13	60→42	73→55	10 / 5
**HA**	9.79	60→42	73→55	5 / 10

Abbreviations. Acetic acid: AA; Formic acid: FA; Propionic acid : PA ; Isobutyric acid : IBA ; Butyric acid : BA ; Butyric- 1,2- ^{13}C2 : BA-LAB ; Propionic d6 acid : PA-LAB ; Sodium acetate : ^{13}C2 AA-LAB ; Sodium Formate- ^{13}C FA-LAB ; Isobutyric acid d6 : IBA-LAB

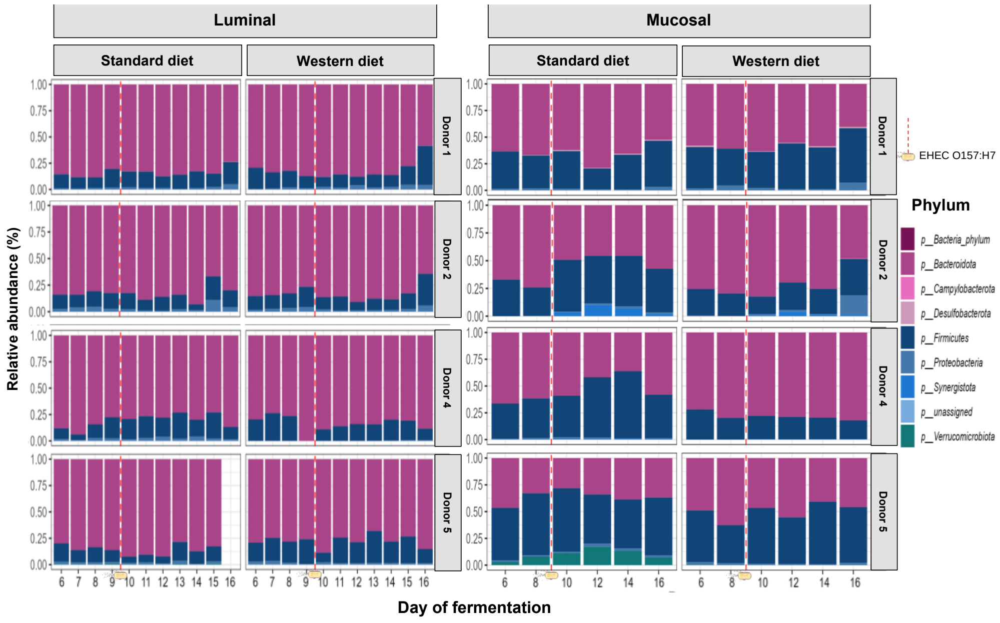
Luminal
Mucosal
Standard diet
Western diet
Donor 1
Donor 2
Donor 4
Donor 5
Relative abundance (%)
Day of fermentation
EHEC O157:H7
Phylum
p__Bacteria_phylum
p__Bacteroidota
p__Campylobacterota
p__Desulfobacterota
p__Firmicutes
p__Proteobacteria
p__Synergistota
p__unassigned
p__Verrucomicrobiota

(a)

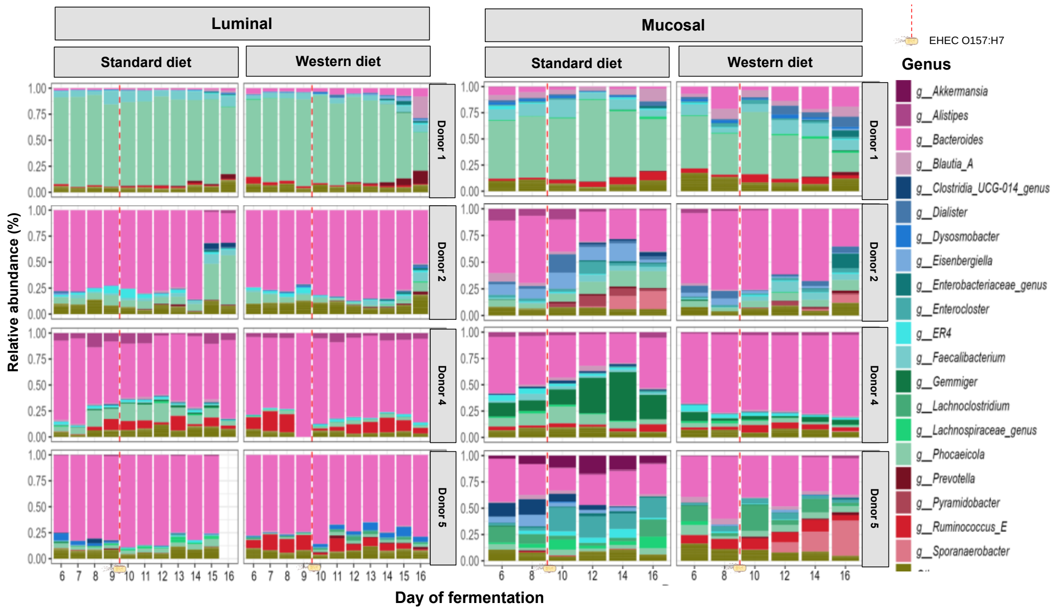
Luminal
Mucosal
Standard diet
Western diet
Donor 1
Donor 2
Donor 4
Donor 5
Relative abundance (%)
Day of fermentation
EHEC O157:H7
Genus
g__Akkermansia
g__Alistipes
g__Bacteroides
g__Blautia_A
g__Clostridia_UCG-014_genus
g__Dialister
g__Dysosmobacter
g__Eisenbergiella
g__Enterobacteriaceae_genus
g__Enterocloster
g__ER4
g__Faecalibacterium
g__Gemmiger
g__Lachnoclostridium
g__Lachnospiraceae_genus
g__Phocaeicola
g__Prevotella
g__Pyramidobacter
g__Ruminococcus_E
g__Sporanaerobacter

(b)

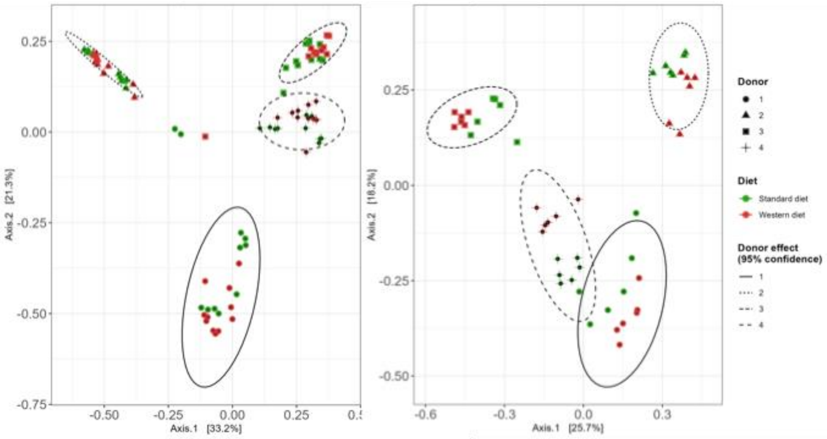
Axis.1 [33.2%]
Axis.2 [21.3%]
Axis.1 [25.7%]
Axis.2 [18.2%]
Donor
Diet
Standard diet
Western diet
Donor effect (95% confidence)

(c)

Figure S1. Effect of diet on microbiota composition in the M-ARCOL challenged with EHEC. Fermentations were run in the M-ARCOL model, every two bioreactors were inoculated with a fecal sample from one of 4 healthy donors (n=4), supplied with either Standard or Western diet, and challenged at Day 9 with EHEC O157:H7 strain EDL933 (dashed line). Samples were taken regularly from both the luminal and mucosal phases of the *in vitro* colon model and microbiota composition analysed by 16S Metabarcoding. Relative abundance of main bacterial populations at the phylum (a) and genus levels (b). (c) PCoA analysis of 16S Metabarcoding results showing donor effect on microbiota composition (95% confidence ellipses).

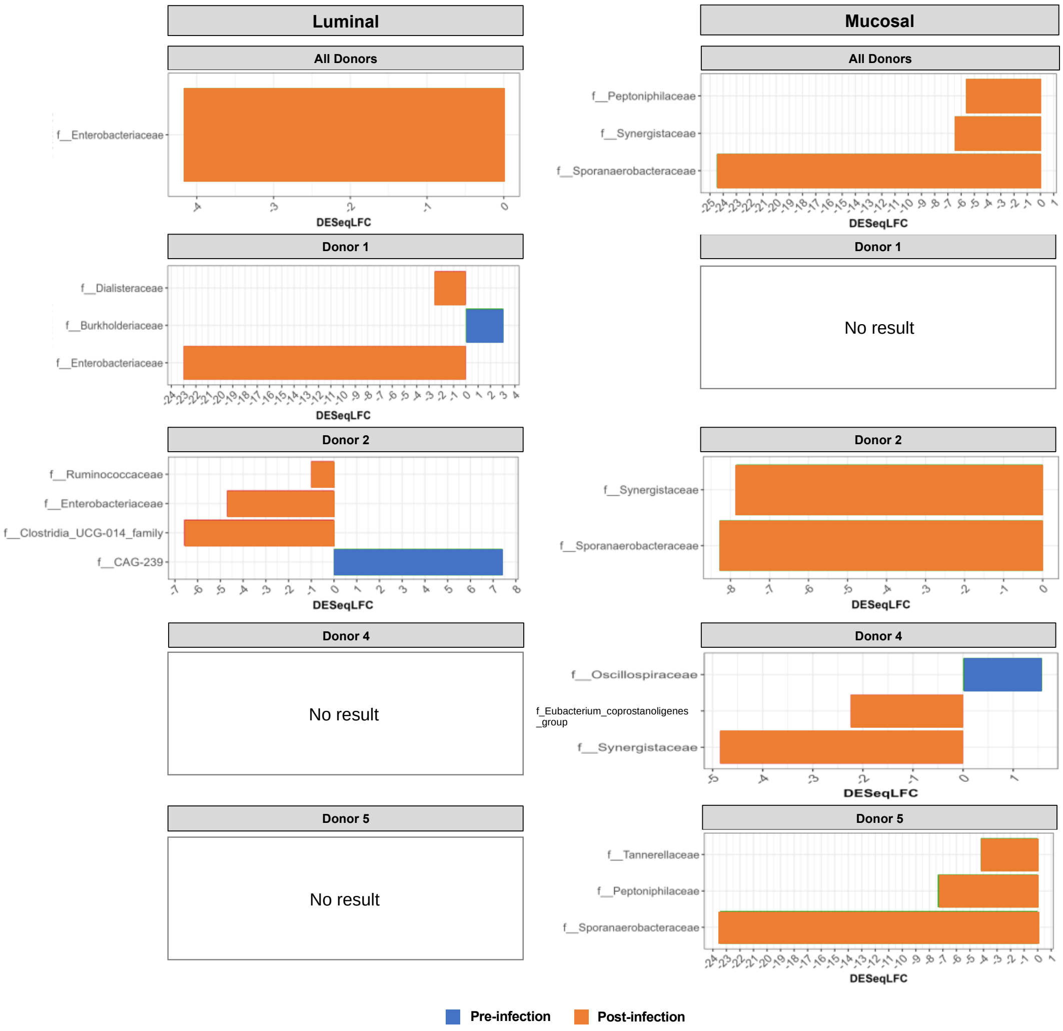

Figure S2. Differential analysis of pre- *versus* post-infection effect on bacterial composition in the luminal and mucosal environments of the M-ARCOL Fermentations were run in the M-ARCOL model, every two bioreactors were inoculated with a fecal sample from one of 4 healthy donors (n=4), supplied with either Standard or Western diet, and challenged at Day 9 with EHEC O157:H7 strain EDL933. Samples were regularly collected in the pre and post-infection periods in both the luminal and mucosal phases of the *in vitro* colon model and microbiota composition analysed by 16S Metabarcoding. Differential analysis were performed with three different methods (DeSeq2, Metacoder and MetagenomeSeq R-analysis). Blue and orange color codes indicate families more abundant in the pre-infection and post-infection phases, respectively. Presented families are differentially more abundant in at least one method.

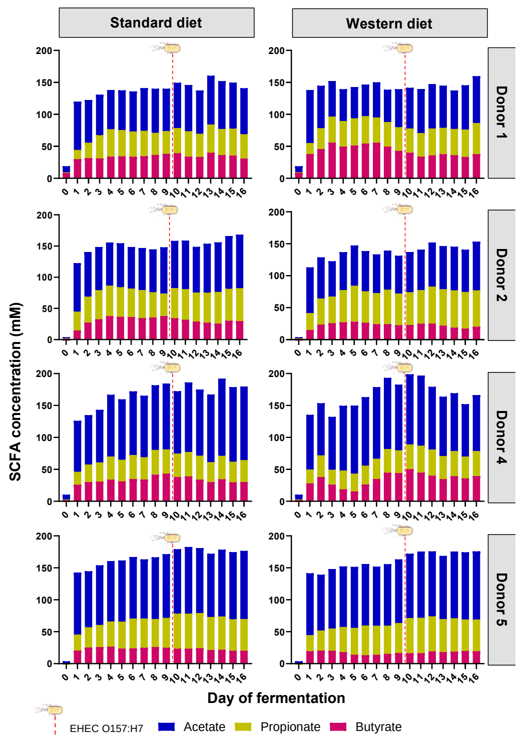

Figure S3. Effect of diet on individual SCFA production in the M-ARCOL challenged by EHEC infection. Fermentations were run in the M-ARCOL model, every two bioreactors were inoculated with a fecal sample from one of 4 healthy donors (n=4), supplied with either Standard or Western diet, and challenged at Day 9 with EHEC O157:H7 strain EDL933 (dashed line). Samples were regularly collected in the luminal medium to determine SCFA production. Results are expressed in mM for each donor.
